# The Influence of Roux-en-Y Gastric Bypass and Diet on NaCl and Sucrose Taste Detection Thresholds and Number of Circumvallate and Fungiform Taste Buds in Female Rats

**DOI:** 10.3390/nu14040877

**Published:** 2022-02-19

**Authors:** Kellie M. Hyde, Ginger D. Blonde, A. Valentina Nisi, Alan C. Spector

**Affiliations:** Department of Psychology and Program in Neuroscience, Florida State University, Tallahassee, FL 32306, USA; kelliemhyde@gmail.com (K.M.H.); blonde@psy.fsu.edu (G.D.B.); nisi@psy.fsu.edu (A.V.N.)

**Keywords:** taste, taste sensitivity, taste thresholds, Roux-en-Y gastric bypass, high-fat diet, taste pores, bariatric surgery, rat, gustatory system

## Abstract

Roux-en-Y gastric bypass (RYGB) in rats attenuates preference for, and intake of, sugar solutions. Additionally, maintenance on a high-fat diet (HFD) reportedly alters behavioral responsiveness to sucrose in rodents in short-term drinking tests. Due to the fact that the behavioral tests to date rely on the hedonic value of the stimulus to drive responsiveness, we sought to determine whether taste detection thresholds to sucrose and NaCl are affected by these manipulations as measured in an operant two-response signal detection paradigm. Female rats were maintained on HFD or chow for 10 weeks, at which point animals received either RYGB or SHAM surgery followed by a gel-based diet and then powdered chow. Upon recovery, half of the rats that were previously on HFD were switched permanently to chow, and the other rats were maintained on their presurgical diets (*n* = 5–9/diet condition × surgery group for behavioral testing). The rats were then trained and tested in a gustometer. There was a significant interaction between diet condition and surgery on NaCl threshold that was attributable to a lower value in RYGB vs. SHAM rats in the HFD condition, but this failed to survive a Bonferroni correction. Importantly, there were no effects of diet condition or surgery on sucrose thresholds. Additionally, although recent evidence suggests that maintenance on HFD alters taste bud number in the circumvallate papillae (CV) of mice, in a subset of rats, we did not find that diet significantly influenced taste pores in the anterior tongue or CV of female rats. These results suggest that any changes in sucrose responsiveness in intake/preference or hedonically oriented tests in rats as a function of HFD maintenance or RYGB are not attributable to alterations in taste sensitivity.

## 1. Introduction

Natural selection has generated a finely tuned gustatory system in vertebrates able to detect, recognize, and differentiate ingestible foods and fluids. As such, taste is a relevant component in the control of ingestive behavior [1,2]. Taste serves at least three partially dissociable adaptive functions [3,4]. First, it enables animals to detect and discriminate among tastes (sensory-discriminative). This helps animals characterize the amount and quality of chemical stimuli in foods and fluids—a function that allows them to unconditionally recognize specific nutrients or learn about their postoral consequences. Second, taste promotes the ingestion or avoidance of certain types of chemical stimuli (motivational/hedonic). This function serves to encourage ingestion of needed nutrients and the rejection of potentially harmful substances. Finally, certain taste stimuli trigger specific digestive and metabolic preparatory responses, collectively referred to as cephalic phase reflexes, which optimize the assimilation of nutrients (physiological). Importantly, all these gustatory functions have the potential to significantly impact dietary choices [1,2].

The complicity of the taste system in the onset and perpetuation of obesity has been under study. Obesity is associated with increased caloric intake and hyperphagia, specifically of calorically dense foods, especially those high in sugar and/or fat [5,6]. Although some aspects of the functional consequences of obesity on physiology and behavior are well known, the relationship between gustatory function and relative body mass composition remains unclear. Studies testing taste function of people with obesity have mostly focused on differences in hedonic responsiveness to a given tastant and not the actual ability to detect its presence. Of the studies that have examined the relationship between taste threshold and BMI, some found positive associations [7,8,9,10], some negative [11,12], and others found no association [13,14,15]. These inconsistencies span a variety of qualitative taste stimuli, have been noted across sex and age groups, and reflect various differences in methodology. Moreover, it is unknown whether obesogenic diets themselves influence sensory-discriminative taste function. That said, some recent studies in mice [16,17] and rats [18] have found that maintenance on a high-fat diet (HFD) can result in a lower number of taste buds in certain oral fields, can blunt taste receptor cell and behavioral responsiveness to sweeteners, and can decrease expressions of key intermediaries essential for GPCR signaling in taste bud cells. Such findings result in the hypothesis that taste sensitivity may be influenced by exposure to diets high in fat. Accordingly, one goal of this study was to test whether maintenance diet influences the taste detection threshold of two salient, common, and nutritionally relevant taste stimuli, sucrose and NaCl, in a rat model.

Additionally, we tested whether Roux-en-Y gastric bypass (RYGB) has an effect on perithreshold taste sensitivity to these tastants. Along with sleeve gastrectomy, RYGB is one of the most effective bariatric surgeries performed [19] and involves a reorganization of the gastrointestinal tract such that most of the stomach and duodenum are bypassed, and ingested nutrients are diverted directly to the jejunum [20]. Some of the hallmark outcomes of this procedure include reduced body weight (15–25% body weight loss up to 20 years), altered gut hormone responsivity, decreased appetite and intake, alteration of the flow and concentration of bile acids, and altered gut microbiota profiles [21,22,23,24,25,26]. RYGB is also sometimes associated with changes in diet choice or taste function in humans. Some studies have found that patients report decreased intake or preference of foods that are high in fat or sugar and increased intake of fruits and vegetables [27,28,29]. However, these nutrient-specific changes are not always reported or are not maintained over time [30,31,32,33].

While there are several postsurgical outcomes that might result in changes in intake and food selection, some evidence supports an effect of surgery on taste [30,31,32,34]. Alterations in “sweet” taste, both objectively and subjectively, are the most commonly reported change [35,36,37,38,39,40,41], although the assessment of the effect of RYGB on thresholds to other tastants have generated mixed results [14,42]. Indeed, the influence of reported changes in taste function on food choice and preference remains unclear. Pepino et al. showed that patients after RYGB initially prefer the same concentrations of sugar solutions as controls but experience decreased palatability upon repeated exposure [38]. Although Bueter et al. showed a decrease in detection threshold of sucrose in patients after RYGB, there was no change in the preferred concentration [37]. Additionally, there is evidence to support an influential role of weight loss alone on responsiveness to tastes, although this weight-loss effect is debated [36,43,44,45]. In summary, the mechanism of any surgically induced changes in taste function, or whether such changes actually occur, has yet to be entirely resolved.

Fortunately, animal models have been invaluable for studying the effects of obesity [46,47] and bariatric surgery [48,49], because dietary experience can be precisely controlled, more invasive manipulations and measures can be conducted, and testing conditions can be consistently maintained in the long term. Although the influence of RYGB in humans on food choice and preference is debated, there seems to be consistent evidence for an effect of surgery on diet choice in rodents. Several studies have shown that rats that receive RYGB surgery decrease intake of high-fat or high-sugar food or drink items and/or increase intake of low-fat/-sugar items [50,51,52,53,54,55,56]. Some groups have found decreased hedonic responsiveness to sucrose following RYGB (i.e., assessed in brief-access tests) [57,58,59], but such a surgery-induced influence on taste-related motivational potency of these stimuli is not universally reported [50,54,56,60]. Accordingly, while it is debatable whether RYGB affects taste responsiveness in the motivational domain, sensory-discriminative taste function has never been studied in animal models of RYGB.

Here, the effects of RYGB or sham surgery (SHAM) on taste detection thresholds for NaCl and sucrose were assessed in rats maintained under three different diet conditions. Additionally, an array of physiological and anatomical effects of diet and surgery were studied, including the effect of diet on the number taste buds in the circumvallate and fungiform papillae because there is evidence to suspect decreases [16]. Overall, neither RYGB nor chronic exposure to a high-fat diet (HFD) had any discernable influence on the taste detection of sucrose. There appeared to be a significantly lower NaCl threshold after RYGB in rats maintained on HFD, but this did not survive the Bonferroni corrected comparison. Moreover, in contrast to what was predicted based on the literature, the fat content of the maintenance diet did not affect the number of fungiform papillae in the anterior tongue or the number of fungiform and circumvallate taste pores, nor did RYGB have any impact on these particular measures of the anatomy of the peripheral gustatory system. These are the first studies, to our knowledge, in which the effects of maintenance diet and RYGB on taste detection thresholds have been psychophysically assessed in a rodent model.

## 2. Materials and Methods

### 2.1. Subjects

Sixty-one adult female Sprague-Dawley rats (Charles River, ~3 months-old) were used. Animals were singly housed in rectangular polycarbonate cages with wood-chip bedding, except for brief stages of surgery and recovery as described below. The vivarium was maintained on a 12:12 light:dark cycle and was temperature and humidity controlled. Animals had ad libitum access to food from the cage lid (during acclimation) then from hanging metal hoppers (during training and testing), and deionized water (dH_2_O) from the cage lid except during surgical recovery, training, and testing in the gustometers, and training and testing for the test meal procedure conducted for the analysis of gut hormones. Details of these experimental phases are described below. Female rats were exclusively used due to the fact that the majority of bariatric surgery patients are women [19,61]. All animal procedures were approved by the Animal Care and Use Committee at Florida State University. Refer to Table 1 for the experimental timeline.

The female rats were not monitored for phase of estrous during any phase of the experiment. Based on the complicated design of the testing protocol, it would be nearly impossible to test every female with each concentration of the stimuli tested on each phase of the estrous cycle.

Some health complications resulted in topical treatments with triple antibiotic and liquid bandage (New-Skin^®^; Cedar Knolls, NJ, USA) and specialized bedding for cases of bumble foot (*n* = 8) or skin lesions (*n* = 1) when necessary, as advised by the attending veterinarian. Several cases of mammary and fatty tumors also developed, most requiring no intervention, but in 4 cases, the animals were removed from the remainder of the experiment.

### 2.2. Diet

Upon arrival, animals were allowed 1 week of acclimation to the vivarium. A subset of rats was then given access to high-fat, high-sugar (HFD) chow diet (Research Diets; 45% Fat, 17% Sugar), while the rest of the animals remained on standard lab chow (PMI; LabDiet 5001) as their maintenance diet for 10 weeks. After the 10-week diet acclimation period, animals underwent Roux-en-Y gastric bypass (RYGB) or SHAM surgery and, once recovered, were weaned onto pelleted chow in three diet conditions. Half of the animals that were maintained on HFD pre-surgically were weaned back onto pelleted HFD, and the other half of the original HFD group was switched to low-fat/low-sugar standard lab chow (13.4% fat/8.9% sugar), referred to as the Diet Change (DC) group. Animals that had access to chow during the diet acclimation period were maintained on chow after surgical recovery and referred to as the Chow group. As such, there were three diet conditions: animals maintained only on standard lab chow (Chow), animals maintained on only HFD diet (HFD), and animals that were switched from HFD to standard lab chow (DC). Some animals had more time on HFD before the diet change (range: 10–14 weeks) due to the surgery and recovery timeline. However, there is no reason to believe that this made a meaningful impact on the behavior. Additionally, as explained below, there were several weeks of training that allowed ample time for acclimation to the new diet for the DC animals.

### 2.3. COVID-19

Restrictions and limitations related to the COVID-19 pandemic interfered with data collection during Phase 2. Animals acclimated to diet received surgery and began training as planned. However, 3 days into training, all experimental procedures were halted (except for animal health checks) due to a university-mandated lockdown related to COVID-19. Training and testing commenced as planned after 5 weeks of a holding period during which time the animals remained on their respective maintenance diets, receiving ad lib food and water. Additionally, after testing NaCl for 3 weeks, testing was interrupted again for 8 days during which animals had ad libitum access to dH_2_O and their respective diet. Testing continued as designed, without additional interruption, for the duration of the experiments. We have no reason to believe these interruptions significantly impacted the results or their interpretation.

### 2.4. Surgery

Rats received no fewer than 10 prophylactic injections of Iron Dextran (Henry Schein^®^, Melville, NY, USA; 2.5 mg/kg, s.c.) once a week before surgery. This was performed to minimize the possibility of RYGB animals developing anemia, a common side-effect in humans [21,62]. After surgery, rats that received RYGB were continued on the iron-treatment schedule; animals receiving SHAM surgery were switched to saline injections (4 mL/kg). Due to the fact that they are not vulnerable to anemia to the same degree as the RYGB animals, the SHAM animals only received iron before surgery and then were switched to saline treatment; this avoided the risk of iron toxicity. Two RYGB females (one from each phase) were switched to a higher dose of iron (5 mg/kg) to combat paleness, a sign of anemia.

At least one week before surgery, all rats were acclimated to the postsurgical housing and diet conditions for one night. This included standard polycarbonate rat cages with a raised wire floor over an absorbable lining (Techboard^®^, Shepherd Specialty Papers, Inc., Richland, MI, USA) and a specialized, homemade gel diet. This gel diet was made in the lab and consisted of gelatin, corn starch, whey protein, corn oil, baby vitamins, and dH_2_O.

The night before surgery, the rats were food deprived and moved to the special housing described above. The following morning, the rats were transported from the vivarium to the surgical suite in clean, empty cages. Anesthesia was induced and maintained with vaporized isoflurane (3–5%/L O_2_). Animals were shaved from pelvis to sternum, and the exposed skin was cleaned topically with an antiseptic solution and saline. An incision was made along the midline of the skin and then in the muscle layer of the abdomen to expose the gastrointestinal tract. Approximately 10 cm aboral from the Ligament of Treitz the jejunum was transected, creating distal and proximal jejunal stumps, which were sutured closed (5-0 PDS II suture). A small incision in the proximal stump of the jejunum was then sutured to a similarly sized incision ~27 cm proximal from coecum in a side-to-side anastomosis (6-0 PDS II suture). The gastric pouch was created by transecting the stomach from the esophagus, ~2 mm distal from the esophageal connection, and the stomach remnant was closed (5-0 PDS II suture). A small incision in the distal stump of the jejunum was connected to the gastric pouch in an end-to-side anastomosis (6-0 PDS II suture). The muscle wall and skin were then separately sutured closed (4-0 Vicryl suture). SHAM surgery was conducted with similar exposure and manipulation of the intestines and stomach, but no incisions were made in the gastrointestinal tract. Instead, at each location where incisions were made in the RYGB procedure, only a few interrupted sutures were placed. All animals were given analgesics (Carprofen, 5 mg/kg, s.c.) and antibiotics (Enrofloxacin, 2.3 mg/kg, s.c.) on the day of surgery and allowed at least 1 h to recover from anesthesia in the surgical suite before returning to the vivarium.

### 2.5. Recovery

RYGB and SHAM animals received analgesics and antibiotics for the 3 days following surgery. Animals were slowly weaned from the gel diet and wet mash (4:1, dH_2_O: powder chow) back onto pelleted chow during 10–18 postsurgical days. RYGB surgery was conducted on 38 females, and SHAM surgery was conducted on 23. The most common difficulties encountered were drooling/difficulty transitioning to pelleted food, diarrhea, and leaking anastomosis. Fourteen rats were removed from the experiment and euthanized because of surgical complications exhibited during recovery (see Table 2 for final group sizes).

### 2.6. Stimuli

Taste detection thresholds for NaCl and sucrose were determined. For each of these tastants, the animals were trained to the highest concentration, then challenged to correctly detect the presence of that stimulus vs. dH_2_O as the concentration was systematically decreased by ~0.33 log_10_ units. NaCl served as the control stimulus given that we had no a priori reason to expect that the surgical or the diet manipulations would impact NaCl detection thresholds. Animals were trained on 0.4 M NaCl, then tested on up to 12 concentrations (0.4, 0.2, 0.1, 0.05, 0.025, 0.01, 0.005, 0.0025, 0.00125, 0.006, 0.003, and 0.0015) vs. dH_2_O. Sucrose was chosen to represent a stimulus nutritionally relevant to both obesity and RYGB (i.e., high-sugar foods or drinks). Sucrose concentrations ranged from 0.6 M to 0.0005 M (0.6, 0.3, 0.15, 0.07, 0.0375, 0.01875, 0.01, 0.005, 0.003, 0.002, 0.001, and 0.0005). Reagent grade NaCl and sucrose (MACRON Fine Chemicals, VWR, Radnor, PA, USA) solutions were made fresh daily using a single source of dH_2_O.

During Phase 1, we attempted to additionally test the animals for detection thresholds for linoleic acid (LA), a polyunsaturated fatty acid that has been used to study “fat taste” in humans and rodents [13,63,64,65,66,67]. For this training phase, LA was dissolved in 5 mM EtOH, and then 0.5% Tween in concentrations ranging from 0.18 mM–0.6 M (v 5 mM EtOH or 0.5% Tween in water). Unfortunately, the animals used here were unable to reliably discriminate between LA, at any concentration, and the solvent, and we were unable to complete a testing array for the fatty acid. We, thus, abandoned the administration of this test for Phase 2 animals.

### 2.7. Apparatus

Taste detection thresholds were measured in 4 specialized operant conditioning chambers called gustometers [68]. Each gustometer consists of a sound attenuating chamber that holds an animal testing cage. The testing cage is made of 3 polycarbonate walls, 1 stainless steel front wall, and a wire mesh floor. The front wall has 3 parallel access slots through which an animal can lick fluid from 1 of 3 balls. The center ball (borosilicate glass) is attached to a mechanical arm that positions the ball in front of the center access slot and allows the sample ball to rotate on its horizontal axis. The sample stimulus is pumped from a reservoir outside of the sound attenuating chamber, through PFTE tubing, and dispensed onto the sample ball as an animal is licking. When a trial is complete, the mechanical arm moves the sample ball away from the access slot to a position where it can be rinsed (with dH_2_O) and dried (with pressurized air) before being repositioned back in front of the access slot for the next trial. Response balls (polyoxymethylene) are fixed on either side of the sample ball. Animals are reinforced with water that is dispensed through a Tygon^®^ tube (Saint-Gobain Performance Plastics, Malvern, PA, USA) through the core of each response ball. A more detailed description of the apparatus can be found in Spector et al. [68].

### 2.8. NaCl Training and Testing

A two-response operant taste detection procedure was used to determine the detection thresholds for NaCl and sucrose [69,70,71]. Animals were required to lick a sample, then make a correct response regarding the presence or absence of the taste stimulus by licking on a response ball. The concentration of the taste stimulus was systematically decreased until the animal could no longer distinguish it from dH_2_O (chance performance, 50%). For these experiments, the animals were water-deprived, and the reinforcement was water, ensuring that it was the sensory-discriminative aspects of the taste stimulus that were guiding responses. Home cage water bottles were typically removed on the Sunday before training or testing week and replaced after the session on Friday. Body weight was measured daily to guarantee that the animals were not overly dehydrated. Animals were given up to 20 mL of supplemental water if their body weight fell below 85% of their ad libitum weight. Since the RYGB animals tended to have a more difficult time recovering and maintaining body weight, all animals were provided with supplemental water every day to stave off extensive weight loss in RYGB animals.

**Figure 1:**
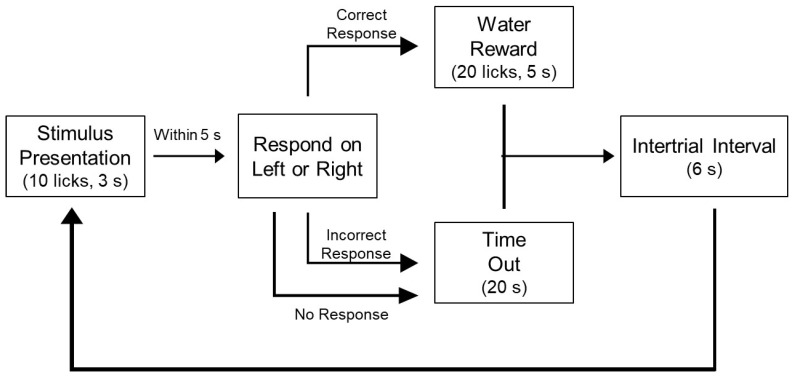
Flowchart of trial parameters. Once a trial had begun, animals had to sample the stimulus and very quickly decide on which side to respond. If they were correct, they received a water reward; if they were incorrect or did not respond, they received a timeout.

Trials were initiated when an animal licked twice within 250 ms on a dry sample ball, after which a preload of ~10 µL was dispensed; for each successive lick, ~5 µL of stimulus was delivered onto the sample ball. Once a trial started, animals could lick for 5 s or 10 licks before the sample ball was moved away from the access slot by the mechanical arm. Animals were allowed 5 s to begin licking on either of the response balls (limited hold). Correct responses were reinforced with 10 s or 20 licks of water, whichever came first. Incorrect responses were punished with a 20-s timeout, during which time no new trials could be initiated. The intertrial interval was 6 s to accommodate the time required to wash, dry, and reposition the sample ball. Sessions were 30 min in duration (Figure 1).

The animals were first trained to receive water from one of three access ports in the gustometers. On a given day during spout training, water-deprived animals licked ad libitum for 30 min from either the right response, left response, or center sample ball while the other 2 balls were blocked by a shutter. This training phase lasted 6–15 days.

Side training was conducted over 6–10 training days, during which animals would lick either 0.4 M NaCl or dH_2_O from the sample ball, and then they received water from the assigned response ball, with access to the other response ball covered by a shutter. Side assignments were counterbalanced between surgery groups and diet conditions. A limited hold was added to session parameters (180 s). This required the animals to respond on one of the reinforcement balls within 180 s of sampling the presented stimulus or a new trial would be initiated. The limited hold was included to encourage animals to respond after the stimulus was tasted. Animals were reinforced with 20 licks of water from the response ball (5 s). No punishment (timeout) was given if no response was made.

Session parameters were changed again for Alternation training to include a 20-s timeout (punishment), during which no trials could be initiated following an incorrect response or no response, and the limited hold was shortened to only 15 s. During this phase, both NaCl and dH_2_O were presented in the same session, one at a time, until animals responded correctly (nonconsecutively) several times, at which point the stimulus was switched. During the 4 training days, the required number of correct responses before a switch decreased from 8 correct responses to 6, 4, and then 2.

Random training occurred in 2 phases. For Random I training, the limited hold was decreased to 10 s, and stimuli were randomly presented in blocks of 4 or 6. Random II training was the similar, but the limited hold was decreased to 5 s. Once all the animals could correctly respond to at least 80% of the trials, testing began. The entire training process took 9 weeks.

The first 3–4 concentrations of NaCl were tested in succession (without stimulus control test sessions) starting with the highest concentration (0.4 M) and decreasing until one or more animals performed at <80% on trials with a response. This was performed for efficiency because, based on prior experience in the lab, we were confident that the concentrations were clearly detectable and there was no need for control sessions. The remainder of the testing sessions was interposed between stimulus control sessions. During a control session, all of the testing parameters were maintained, and a single stimulus concentration at which an individual animal had previously performed with 80% or greater accuracy was tested against water. On very rare occasion, animals were given the wrong control concentration; however, careful review of the analyses revealed no meaningful effect on the conclusion or interpretation of the behavior. In general, animals were deprived of water on Sunday evenings, stimulus control sessions were given on Monday, Wednesday, and Friday, and testing sessions were Tuesday and Thursday. Water was returned after the control session was completed on Fridays. The concentration of NaCl was systematically decreased across testing sessions by ~0.33 log_10_ units until an animal was performing near 50% (chance) accuracy. We do note that, in the initial experimental design, we intended to run all animals to the overall lowest concentration of a given stimulus but became concerned about extinction causing a loss of stimulus control and decided to stop testing animals once 50% performance was achieved, on an individual basis. Before this change, some of the rats were additionally run on 0.07 mM NaCl after they had reached near 50% performance. In an effort to standardize this criterion across all animals, the performance at 0.07 mM NaCl was not included in the analyses.

### 2.9. Sucrose Training and Testing

Following NaCl testing, the animals were given one-week off. Animals were then started on the Random II protocol (see *NaCl Training and Testing*) with 0.6 M sucrose as the stimulus, which lasted 3–5 days. Testing for sucrose started with a stimulus control day (0.6 M Sucrose) followed by a test day at the same concentration; the testing schedule then continued with test days (on Tuesday and Thursday), flanked by control sessions (on Monday, Wednesday, and Friday). Sucrose concentration decreased by ~0.33 log_10_ units on each test day (0.6 M–0.0005 M sucrose). Testing continued until each animal’s performance dropped to near 50% (chance).

### 2.10. Stimulus Control Test

At the end of taste detection threshold testing, a stimulus control test was completed. This single day test followed the same testing parameters as the detection tests, but each of the trials presented the same solution (0.5% Tween (Phase 1) or dH_2_O (Phase 2). Here, however, half of the trials were arbitrarily assigned as “taste” and half were assigned as “solvent.” As such, animals that were under stimulus control should perform at chance (50%), suggesting the animal was not relying on extraneous, nonchemical cues to guide performance in the detection task.

### 2.11. EchoMRI

Following taste detection testing, female rats (Phase 2) were assessed for body composition (EchoMRI^TM^-500; EchoMRI, Shanghai, China). Animals were scanned nondeprived. Bone mass was calculated by the difference of fat mass, lean mass, and free water from body weight on the day of the scan. Fat mass, lean mass, and bone mass are all presented as a percentage of the body weight on the day of the scan. Unfortunately, the animals from Phase 1 were tested before the arrival of the EchoMRI machine. Therefore, only rats from Phase 2 were included in the analysis of EchoMRI body scans.

### 2.12. Hormone Analysis

In addition to the behavioral assays, postprandial levels of glucagon-like peptide 1 (GLP-1) and leptin were assessed. GLP-1 is an incretin hormone released from intestinal L-cells and is associated with appetite and satiation [72]. Patients and rodents experience increased postprandial release of GLP-1 following RYGB surgery [21,73], and the level of hormone response, in humans, can predict the success of surgery regarding weight loss [74,75]. Leptin, released from adipose tissues, also has a functional role in regulating food intake and has a known association with obesity [76,77]. As a validation of the RYGB model (GLP-1) and a proxy for body adiposity (leptin), rats were tested for postprandial GLP-1 response and plasma leptin.

Animals were deprived of food for 23 h, then given 30-min ad libitum access to chocolate Ensure^®^ (Abbott Laboratories, Abbott Park, IL, USA) in their home cage for 3 training days. On the third training day, food was returned for a 4-night break. On the afternoon of the 7th day, animals were deprived of food again. A warm-up session was provided the day prior to test day during which food-deprived animals received 30-min of ad libitum access for assurance. The following day, animals were again given a 30-min ad libitum test, followed immediately by a 30-min holding period before being moved to a perfusion room for cardiac puncture. For the puncture, animals were deeply anesthetized with a euthanasia agent containing sodium pentobarbital (>250 mg/kg, intraperitoneal), the heart was exposed through thoracotomy, and ~3 mL of blood was withdrawn from the heart. Blood was immediately stored in K3 EDTA tubes on ice until centrifuged. Plasma was taken from each sample, aliquoted, and stored in centrifuge tubes at −80 °C until used. Enzyme-Linked Immunoassays (ELISA) were conducted for GLP-1 and leptin (both from Millipore Sigma, Burlington, MA, USA) according to manufacturer’s instructions.

### 2.13. Collection and Analysis of Fat Pads

As an additional measure of body adiposity, gonadal, retroperitoneal, and perirenal fat pads were collected and weighed (Phase 2). Following cardiac puncture (see *Hormone Analysis*), the descending aorta was clamped a few millimeter rostral to the diaphragm to preserve the integrity of the fat pads and then transcardial perfusions were performed with 4% paraformaldehyde (PFA). Of the animals included in fat pad analysis, 3 females received full-body perfusions due to a misplaced or loose clamp. However, the fat pads of these animals were similar to others in respective groups; thus, we do not believe that the perfusions significantly compromised the weight of the fat pads, and no adjustments were made in the analysis.

### 2.14. Histology

**Figure 2:**
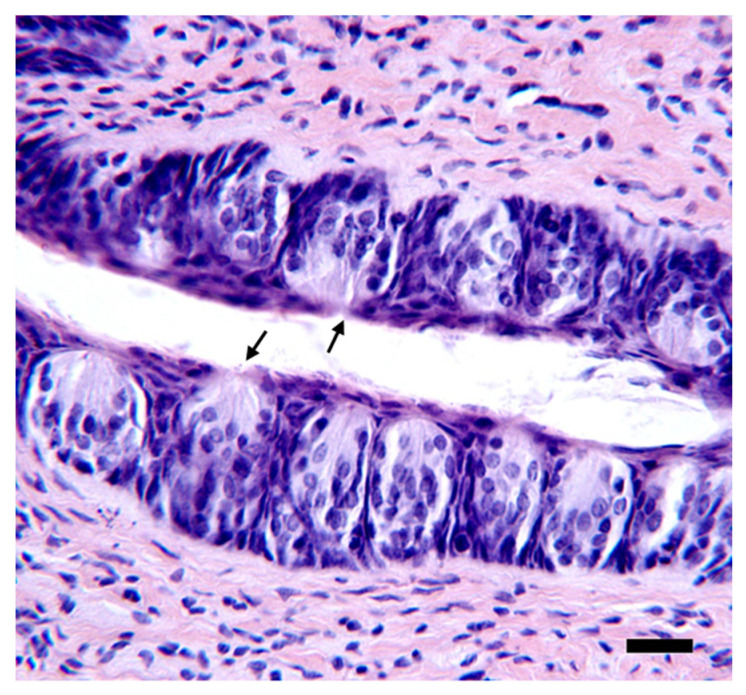
Representative image of CV Slice. A representative image showing the morphology of a pore (black arrows). Photomicrograph adjusted for brightness and contrast. Scale bar represents 25 µm.

Following the perfusion, tongues were removed and stored in PFA. Taste pores were quantified from the fungiform papillae of the anterior tongue and from the circumvallate (CV) papillae of the posterior tongue. The anterior portion of the tongue was separated from the posterior portion and saturated with 0.5% methylene blue then dipped in dH_2_O to remove excess stain. Most of the musculature from the anterior portion of the tongue were removed, and the epithelium, remaining muscle, and connective tissue were pressed between two glass slides. This technique allows the clear identification of pale-staining fungiform papillae intermixed among the stained non-gustatory filiform papillae under a light microscope. Taste pores, which were counted, were identified as a blue dot roughly centered in the fungiform papillae, which were also counted. The taste pore is considered a proxy of a morphologically intact taste bud [78]. Posterior tongue samples were embedded in paraffin, sliced on a rotary microtome (10 µm), and mounted on glass slides. These samples were stained using hematoxylin and eosin, and the taste pores of the CV were quantified under a light microscope (Figure 2). In some cases, CV tissue sections could not be accurately examined normally because of the inadvertent overlap of trench walls. To account for slices that could not be counted, a correction factor was applied. For individual samples, the total number of countable pores was quantified and divided by the number of countable slices encompassing the CV. This factor was then multiplied by the total number of damaged slices and added to the number of taste pores counted from intact CV sections. In essence, we were accounting as accurately as possible for the missing sections by using the average number of pores per section based on CV slices that could be accurately counted for an individual animal. Furthermore, we only conducted statistical analysis on samples containing 50% or more of intact CV slices. A single individual (KMH) counted the taste pores of the anterior tongue and CV and was blind to the diet and surgical condition of the samples until taste bud quantification was completed.

### 2.15. Data Analysis

For detection testing, individual animal performance on trials with a response was collapsed over test and control days giving an average proportion correct for each concentration of a given stimulus tested for each animal. Curves were fit for each animal using a logistic function:

$$f\left(x\right)=\left[\frac{a-0.5}{1+10^{\left(log_{10}x-c\right)b}}\right]+0.5$$

where *x* represents stimulus concentration, *a*, the performance asymptote, *b*, the slope of the curve, and, *c*, the log_10_ of the stimulus concentration at ½ asymptote (EC_50_). The concentration at ½ asymptote is the inflection point on the curve and, thus, is a sensitive measure of the location of the psychometric function and was, thus, operationally defined as the threshold concentration.

In general, animals were run Monday–Friday, with testing on Tuesday and Thursday flanked by control sessions (Mon, Wed, and Fri). On an individual basis, health circumstances required some instances when an animal was only run 3 days during that given week (i.e., control, test, and control). Additionally, in some instances, external factors prevented an animal from performing optimally for a given test or control session. This most often resulted from machine malfunctions. To the best of our ability, assessments were made regarding these factors, which sometimes resulted in a repeated test or control session. Missing data (*n* = 5) or data, which were obviously inaccurate based on individual performance (*n* = 2), were replaced by taking the average performance from the test concentration higher and the concentration lower than the missing point. These instances account for ~0.6% of the detection data analyzed.

Moreover, a correction was made to adjust for an error made in making the 0.01 M sucrose solution. Animals in Phase 2 were run on 0.009 M sucrose initially, and even though this discrepancy was small, the rats were retested at the correct concentration (0.01 M) at the end of sucrose testing for consistency. Finally, animals in each phase were tested until individual performance reached chance levels based on binomial analyses; by design, some animals were tested 1 or 2 concentrations lower than others. This was performed to prevent extinction in animals that had clearly reached their limits of stimulus detectability. In order to account for missing data at the bottom of the curves, the proportion correct for the lowest concentration on an individual basis was extended and applied to the lowest concentration run overall. Performance of the animals was averaged at each concentration (including extrapolated data) and used to generate mean curves for each diet and surgical group. In order to assess differences between groups on each of the three parameters of psychometric functions (asymptote, EC_50_, and slope), we used two-way ANOVA. Paired comparisons were used to dissect significant interactions or main effects of diet or surgery and were conducted using t-tests applying Bonferroni corrections when appropriate. In order to guard against being overly conservative in our paired comparisons, the uncorrected *p*-values are reported but are bolded if they survived Bonferroni correction.

Comparisons of body weight and percent change in body weight were confirmed with ANOVA. During Diet Acclimation, the body weights of each animal on a given Monday were used to determine the group means for that Acclimation Week. Individual body weights during this phase were compared each week to the body weight on the first day animals received acclimation diet. That is, individual body weights from each successive week during the diet acclimation period was compared to the individual weights on the first day of diet acclimation to determine percentage change. Changes in body weights for the duration of the experiment were assessed using the ad libitum body weight of individual animals before water deprivation for each week of testing. The percentage change in body weight during testing was calculated as weekly percentage change on a given Sunday (before water deprivation) from the ad lib body weight of an individual animal the day before surgery. Of note, animals in Phase 1 were trained for 10 weeks (10 training body weights), while animals in Phase 2 were trained for 9 weeks (9 body weights). To standardize statistical comparisons, only body weights from the first 9 weeks of training were used in analyses during the training phase. We do not believe this made a meaningful interpretive impact. Statistically significant group differences in taste pore counts were determined by two-way ANOVAs and paired comparisons where applicable. Due to exceptionally low group sizes in the RYGB HFD group for fat pad measures and hormone tests, this group was excluded from statistical analysis of those measures.

Pearson’s correlations were conducted between taste thresholds and fat mass, hormones, and taste pores. Additional correlations were conducted for key measures from hormone and body composition tests. All of these correlations were performed for all of the groups collapsed as well as within each surgical condition. We did not attempt to correct *p*-values for these correlations.

The conventional *p* ≤ 0.05 was considered statistically significant. Health complications and tissue loss caused group sizes to fluctuate for each of our statistical analyses. Our best efforts were made to include as many animals as possible from each group in each analysis (see Table 2).

## 3. Results

### 3.1. Diet and Surgical Influence of Body Weight and Fat Mass

**Figure 3:**
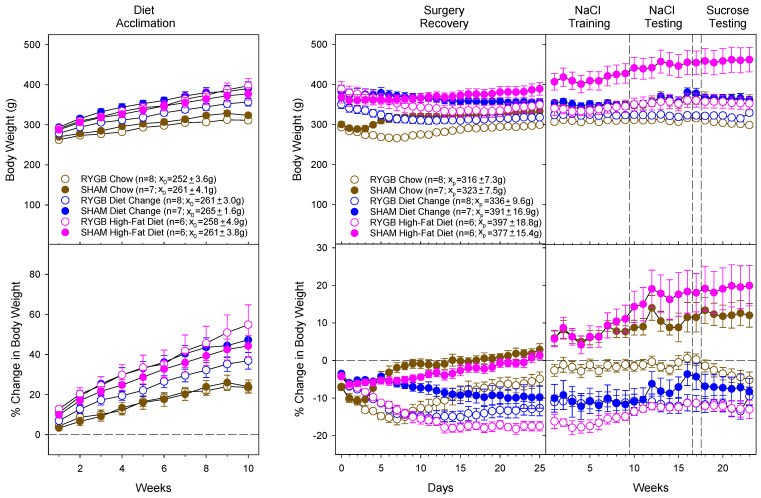
Mean (±SE, standard error) of body weight (**top** panels) and percentage change in body weight (**bottom** panels) of female rats during each of the critical periods. For the left panels, the mean body weight for each diet group on the first day of diet acclimation served as baseline (x_0_; mean ± SE; **left**) for percentage change analysis during the Diet Acclimation period. Week 1 reflects the change in body weight from the first day of the diet acclimation period. In the right panels, mean body weight on the day before surgery served as baseline (x_p_; mean ± SE; right) for percentage change comparisons for the remainder of the experiment. Day 0 reflects the food-deprived body weight of animals on the day of surgery. RYGB animals are represented by open circles and SHAMs by filled circles. Brown symbols represent the Chow group; blue, the diet change; pink, the high-fat diet.

Analysis of body weights is presented as daily (Surgery and Recovery only) or weekly body weight (Table 3) or changes (percentage change; Table 4) in body weight (Figure 3). During acclimation, percentage change was found by analyzing weekly changes from the first day of the diet acclimation period during recovery and detection training and testing, from the day before surgery (which also serves as the final ad libitum day before diet switch for DC animals). The surgical group designation for the acclimation period (before surgery) was based on the surgery each animal would eventually receive.

As expected, HFD exposure (in the HFD and eventual DC groups) drove significant increases in body weight and percentage change during the acclimation period compared to the rats maintained on chow. Moreover, following surgery, RYGB promoted significant decreases in body weight relative to each respective SHAM diet condition. Significant decreases in weight were also noted in the SHAM DC group relative to both the HFD and Chow SHAM animals. Effects of diet and RYGB on absolute weight and changes in weight were observed throughout the experiment, primarily driven by weight gain in the HFD SHAM animals and lower body weights of the animals with RYGB in each diet condition.

**Figure 4:**
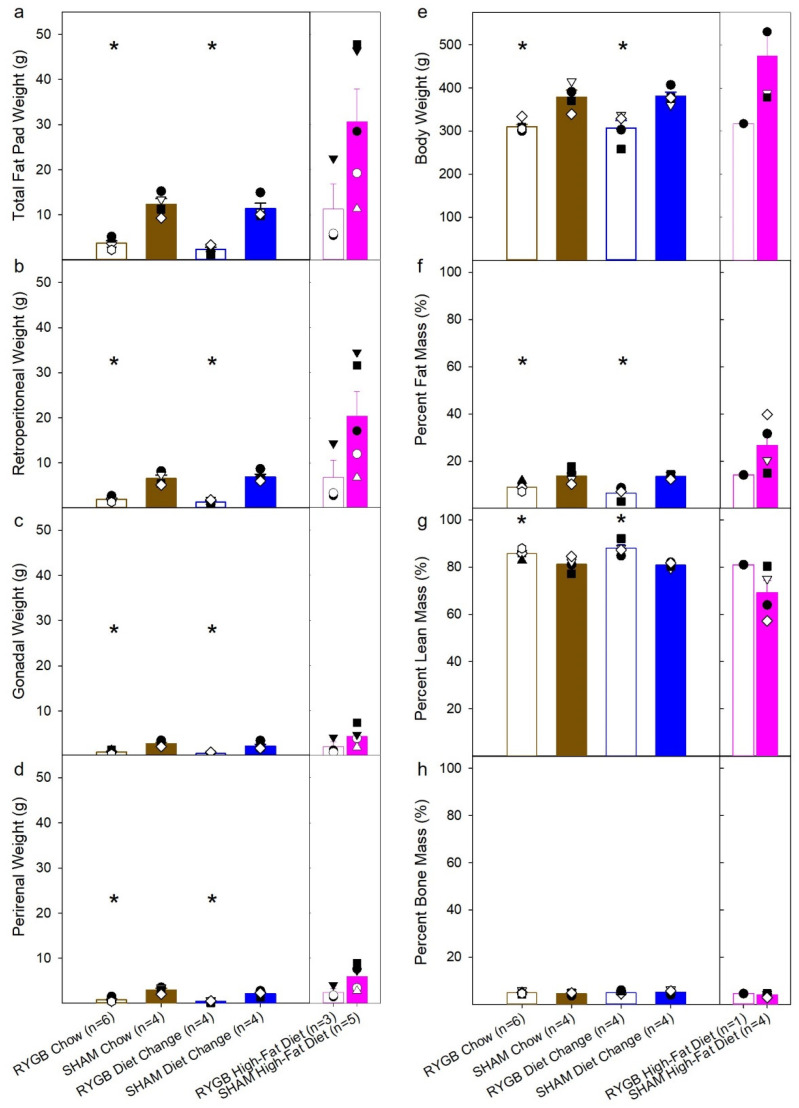
Mean (± SE, standard error) and individual animal data for total fat pad weight (**a**) and weights of the retroperitoneal (**b**), gonadal (**c**), and perirenal fat pads (**d**); the mean (± SE) of the body weight on the day of the EchoMRI scan (**e**); and the percent fat (**f**), lean (**g**), and bone (**h**) mass from the animals in Phase 2. Diet groups are represented as brown for the Chow group; blue, the diet change; pink, the high-fat diet. RYGB group means within each diet condition are represented by open bars, SHAM by filled bars. Due to low group size, the HFD RYGB group could not be included in the statistical analyses and is represented in a separate panel along with its sham-operated control group, which was included in some analyses. Significant differences from paired comparisons (that survived Bonferroni corrections) between the surgical groups within a diet are represented with “*”.

Two measures of body composition were also analyzed. Following threshold testing in Phase 2, the rats were subjected to an EchoMRI, and fat pad weights of the retroperitoneal, gonadal, and perirenal fat pads were also collected (Figure 4, Table 5). For the interpretation of these data, it is critical to acknowledge that these statistics must be viewed with caution because of the low sample sizes (see Table 2). As such, only between-group comparisons for Chow, DC, and SHAM conditions were conducted. Total fat pad weight (Figure 4a), and the individual weights of the retroperitoneal (Figure 4b), gonadal (Figure 4c), and perirenal (Figure 4d) fat pad weights were significantly decreased by RYGB in the Chow and DC groups (p’s < 0.008), but there was no difference between RYGB DC and RYGB Chow groups (p’s > 0.096). Additionally, there were significant effects of diet among the three SHAM groups on total, retroperitoneal, and perirenal fat pad weights (Table 5), but in paired comparisons there was only a trend for HFD animals to have greater values compared with the DC and Chow groups likely because of insufficient power. Analysis of the body weights on the day of the EchoMRI scans (Figure 4e) revealed that the RYGB animals in the Chow and DC groups weighed significantly less than their SHAM counterparts and did not differ between each other (Table 5). There was also no main effect of diet on body weight among the three SHAM groups (Table 5). RYGB animals did have significantly lower percent fat mass (Figure 4f), and greater percent lean mass, compared to their SHAM counterparts in the DC and Chow groups and did not differ between each other (Table 5). An ANOVA indicated a significant main effect of diet for the three SHAM groups on both percent fat mass and percent lean mass. An inspection of Figure 4 suggests that this was primarily driven by differences between the HFD group and the other diet groups (Table 5). However, none of the paired comparisons were statistically significant; this is likely a consequence of low power. There were no differences between the groups on percent bone mass.

### 3.2. Effects of Diet and Surgery on Taste Dectection Thresholds

**Figure 5:**
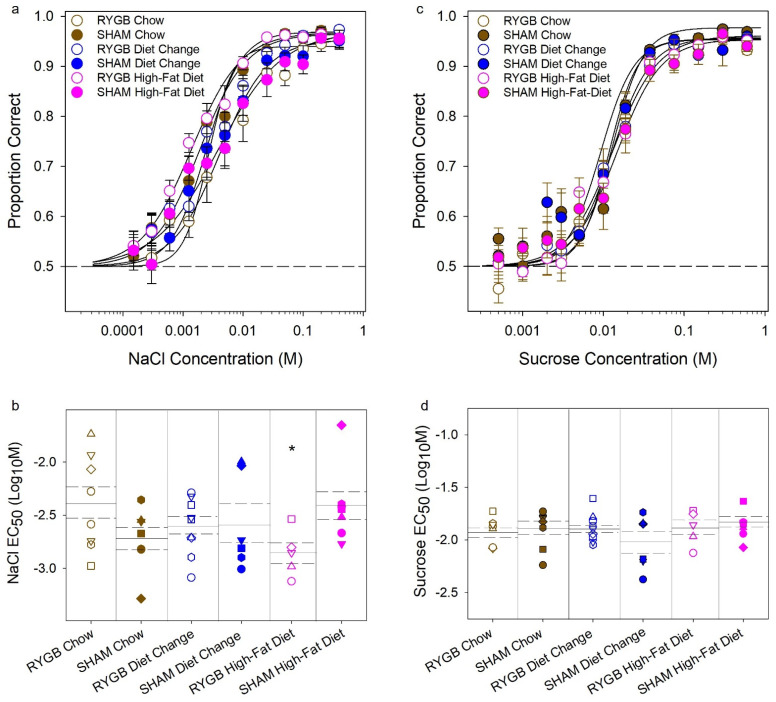
Mean (± SE) proportion correct as a function of stimulus concentration for NaCl (**a**) and sucrose (**c**) for each of the groups. Curve fits reflect group averages for individual data based on the 3-parameter logistic function described in ***Data Analysis***. Individual EC_50_ values for NaCl (**b**) and sucrose (**d**) were also calculated using this logistics function. Means for each diet condition are noted by the solid line; SE, dashed lines. RYGB animals are represented by open circles and SHAMs by filled circles. Brown symbols represent the Chow group; blue, the diet change; pink, the high-fat diet. “*” reflects a significant effect of surgery within a given diet condition as assessed by paired comparison; no comparisons survived Bonferroni corrections.

The groups were compared on the parameters from the logistic function used to generate the curve fits of performance in the NaCl and sucrose detection tasks. Final group sizes are listed in Table 2, thresholds and the mean curves can be found in Figure 5, and group means for the parameters can be found in Table 6. Two-way (surgery × diet) ANOVA revealed no significant effects of diet and surgery, nor a significant diet by surgery interaction on the asymptote or slope of the NaCl functions or the asymptote, slope, or EC_50_ (threshold) for sucrose functions (Table 7).

**Figure 6:**
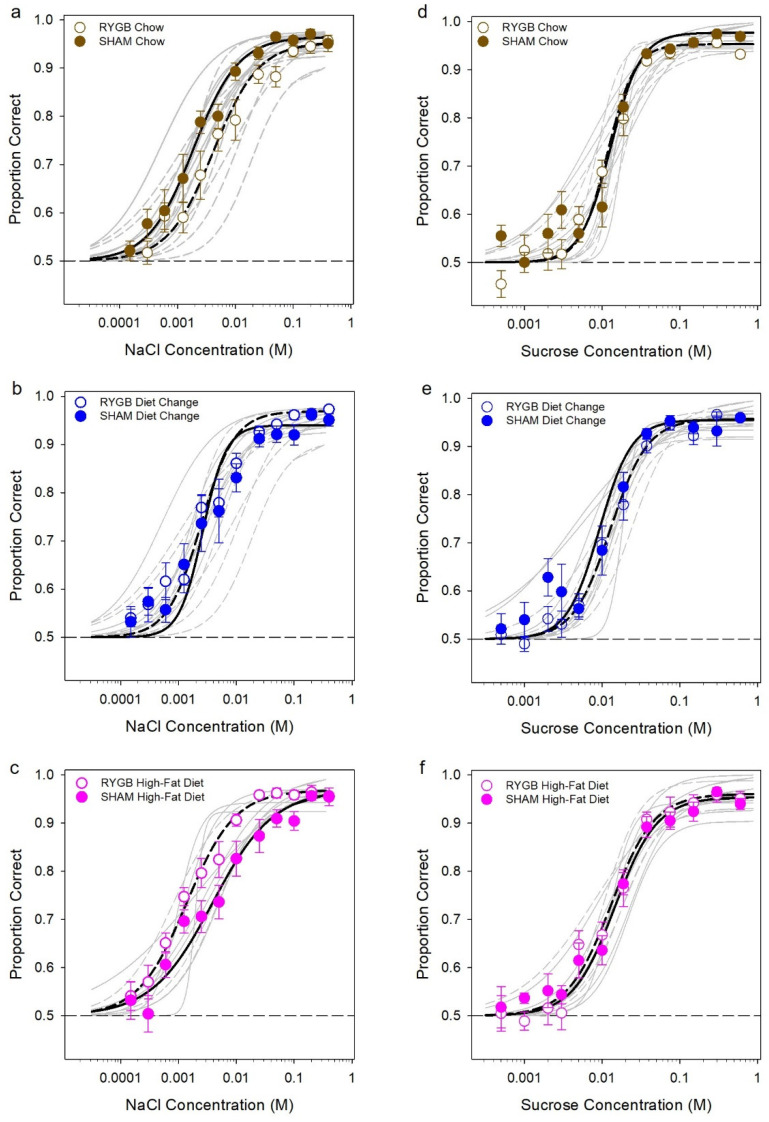
The effect of surgery for each diet condition. Mean (±SE, standard error) proportion correct as a function of stimulus concentration for NaCl (**a**–**c**) and sucrose (**d**–**f**); Chow groups in the top row, DC in the middle row, and HFD in the bottom row. Solid line on curve fits reflects group averages; the grey lines are for individual data based on the 3-parameter logistic function described in ***Data Analysis***. RYGB animals are represented by open circles and dashed lines and SHAMs by filled circles and solid lines. The horizontal dashed line represents chance performance in this task.

We did find a significant surgery by diet interaction for NaCl EC_50_ due to a surgery effect in the HFD group (Table 7); however, no significant differences between the groups survived Bonferroni corrections. Moreover, it is important to exercise caution because the effect of diet may be exaggerated by one outlier in the SHAM HFD group. Additionally, it should be highlighted that there are no differences between the three SHAM groups or between the three RYGB groups. The effect of surgery for each diet condition on individual animal performance across concentrations can be observed in Figure 6 (a–c for NaCl threshold testing; d–f for sucrose).

**Figure 7:**
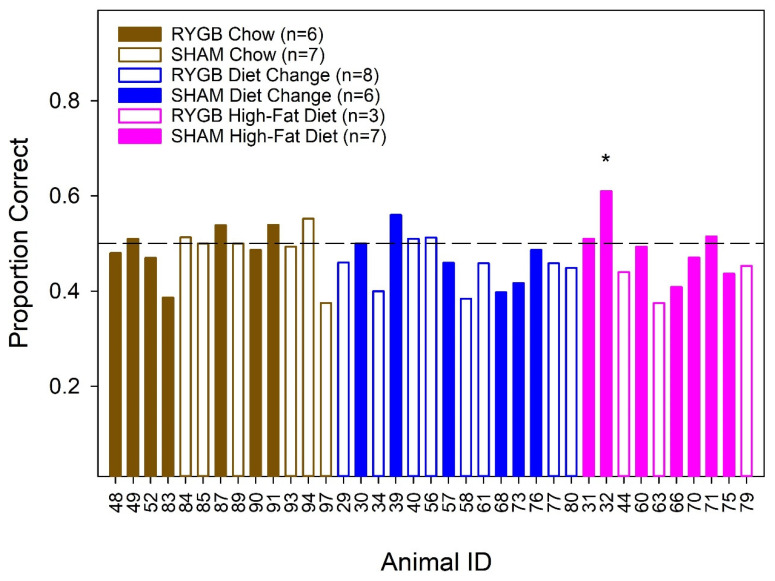
Individual animal performance from the stimulus control test. Filled bars reflect SHAM females and open bars represent the RYGB animals. Brown shading or outlining represent the Chow group; blue, the Diet Change; pink, the High-Fat Diet. One-tailed binomial analyses were used to test for differences greater than chance, represented as significant by “*”. Although the performance of Rat 32 was significantly greater than chance, it was quite poor, and the difference did not survive Bonferroni correction. The horizontal dashed line represents chance performance in this task.

At the end of detection testing, animals completed a stimulus control test (Figure 7). During this test, all reservoirs were filled with the solvent (dH_2_O or Tween) and half of them were arbitrarily assigned as the taste stimulus and the other half as the solvent. Animals that are under stimulus control and, thus, are relying solely on the unique chemical properties of the stimulus to drive responding, should perform at chance (50%). The rats from Phase 2 were tested with dH_2_O; however, rats from Phase 1 were tested with 0.5% Tween. This was conducted because, in Phase 1, after sucrose testing but before the stimulus control test, an unsuccessful attempt was made to assess detection thresholds to the polyunsaturated fatty acid and linoleic acid, which was dissolved in Tween. It is assumed that the different solvents have no impact on the interpretation of the outcomes of the stimulus control test. A few animals were removed early from the study (*n* = 5) and did not complete a stimulus control test. However, based on individual concentration-dependent performance in these cases, we are confident that those rats were under stimulus control and were relying exclusively on chemical cues when detection thresholds were analyzed. One-tailed binomial tests revealed that all animals were under stimulus control after Bonferroni corrections were made.

### 3.3. Quantification of Taste Pores in the Anterior Tongue and Circumvallate Papillae

Recent evidence suggests a decrease in the number of the fungiform papillae in the anterior tongue in humans and the number of taste buds in the anterior tongue and the CV papilla (posterior tongue) in rodents [16,18,79], as well as a decrease in taste bud renewal [17] that appears to be associated with obesity or diet-induced inflammation. Given these findings in the literature, a number of taste pores in the anterior tongue and the CV were quantified and compared across groups.

**Figure 8:**
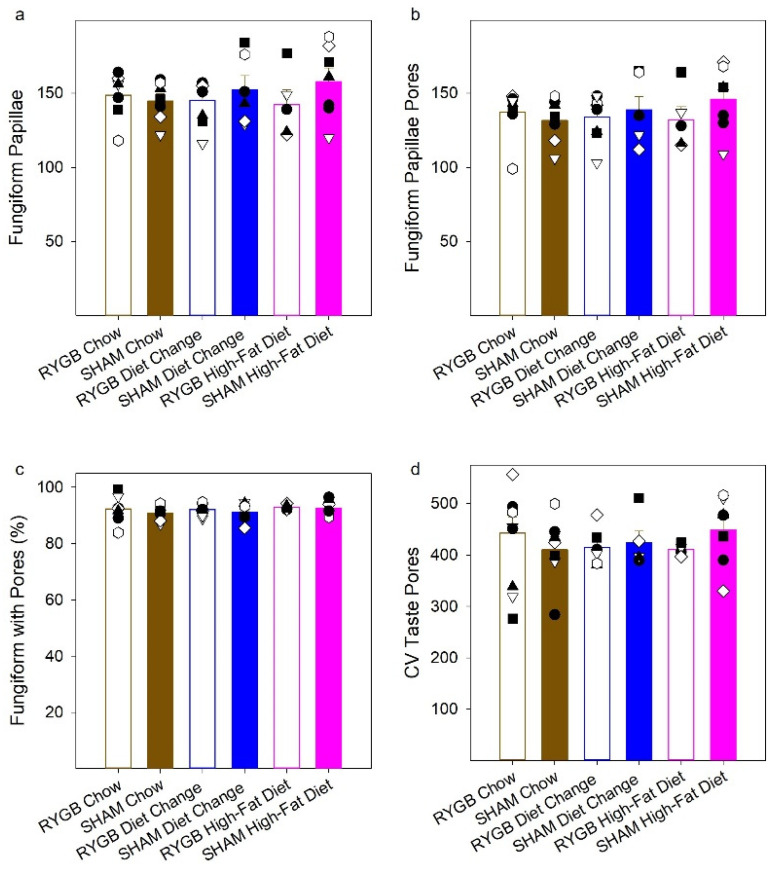
Histological analyses. Mean (± SE) and individual number of fungiform papillae (**a**), number of taste pores in the anterior tongue (**b**), percent of fungiform papillae that had a taste pore (**c**), and number of taste pores in the circumvallate papillae (**d**). RYGB groups are in open bars, SHAMs, in filled. Diet groups are represented as brown for the Chow group; blue, the diet change; pink, the high-fat diet.

There were no effects of surgery (F(1,35) = 1.05, *p* = 0.31), diet (F(2,35) = 0.10, *p* = 0.90), nor a surgery by diet interaction (F(2,35) = 0.89, *p* = 0.42) on the total number of fungiform papillae (Figure 8a). Additionally, a two-way (surgery × diet) ANOVA did not reveal any significant main effects (surgery: F(1,35) = 0.56, *p* = 0.46; diet: F(2,35) = 0.19, *p* = 0.83) or interaction (F(2,35) = 0.95, *p* = 0.40) on the number of taste pores in the anterior tongue (Figure 8b) or on the percentage of fungiform that had taste pores (surgery: F(1,35) = 0.86, *p* = 0.36; diet: F(2,35) = 0.57, *p* = 0.57) or interaction (F(2,35) = 0.093, *p* = 0.91; Figure 8c). Finally, we found no effects of surgery (F(1,30) = 0.055, *p* = 0.82), diet (F(2,30) = 0.073, *p* = 0.93), or interaction (F(2,30) = 0.96, *p* = 0.40) on taste pores in the CV of female rats (Figure 8d).

### 3.4. Hormone Response following a Liquid Test Meal

**Figure 9:**
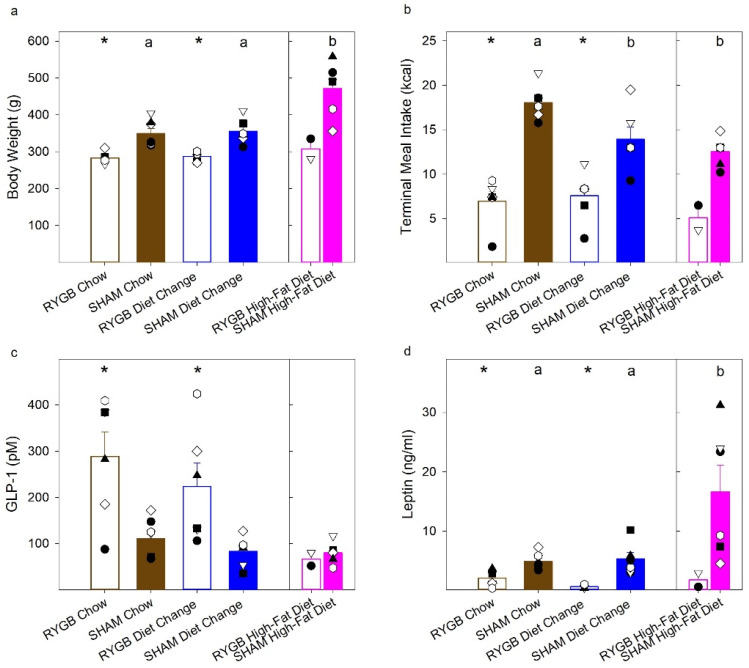
Terminal meal and hormone analyses. Mean (±SE, standard error) of the body weight on test day (**a**), total caloric intake (**b**), postprandial GLP-1 response (**c**), and plasma leptin levels (**d**). Significant differences between SHAM groups found from uncorrected paired comparisons are reflected by different black letters above the bars; “*” represents differences between surgical groups in a given diet condition. There were no differences among the SHAM diet conditions for GLP-1 (**c**); differences between total meal intake (**b**) in the Chow and DC SHAM groups did not survive Bonferroni corrections; surgical differences between the DC group for GLP-1 (**c**) and leptin (**d**) levels did not survive corrections. RYGB groups are in open bars, SHAMS in filled. Diet groups are represented as brown for the Chow group; blue, the diet change; pink, the high-fat diet.

Total plasma GLP-1 and leptin were analyzed following a liquid test meal (Figure 9). Due to the low sample size (Table 2), the RYGB HFD group was excluded from the statistical analyses. Paired comparisons (Table 8) revealed that the RYGB animals in the DC and Chow groups had significantly lower body weight on test day and consumed significantly fewer calories in the test meal relative to their SHAM counterparts. Despite the difference in body weight and caloric intake, GLP-1 levels were significantly higher, and leptin was significantly lower in the RYGB Chow compared to the SHAM Chow group (Table 8). Similar results were found for RYGB DC vs. SHAM DC comparisons, but they did not survive Bonferroni corrections. For each comparison, there was no diet-induced difference between RYGB DC and Chow groups (Table 8).

Comparisons among three SHAM diet conditions (“ANOVA” in Table 8) and paired comparisons between each diet condition for the SHAM groups revealed that HFD SHAM rats weighed significantly more on test day than the DC and Chow groups, which, in turn, did not differ (Table 8). The SHAM DC and SHAM HFD groups consumed the same amount of calories in the test meal (*p* = 0.39), while SHAM Chow animals drank significantly greater calories compared to the SHAM HFD animals, but their intake did not differ from the SHAM DC after Bonferroni corrections. Despite differences in test meal intake among the SHAM groups, there was no influence of diet on GLP-1 response. Finally, the ANOVA across SHAM groups indicated there was a main effect of diet on leptin levels. Paired comparisons indicated that the HFD had significantly increased leptin relative to the DC and Chow groups, but comparisons between the DC and HFD group did not survive Bonferroni corrections (Table 8).

### 3.5. Correlations between Key Experimental Measures

We found the expected positive correlations (Figure S1) between leptin and fat mass as assessed by fat pad weight and EchoMRI. There was also a high positive correlation between fat pad weight and percent fat mass as assessed by EchoMRI, providing cross-validation of both techniques for assessing body adiposity. Fat mass was significantly negatively correlated with GLP-1 and driven by the RYGB group. Additionally, we found some interesting correlations within the surgical groups. For SHAM animals, there was a significant negative correlation between GLP-1 response and NaCl EC_50_. We also found that the NaCl EC_50_ was correlated with CV taste pore numbers. Within the RYGB group, there was a significant correlation between the intake in the terminal test meal and GLP-1 response (see these and other correlations in Tables S1 and S2). For the most part, the significant correlations were modest, accounting for no more than 25% of the variance.

## 4. Discussion

### 4.1. Maintenance on HFD Does Not Affect Liminal Taste Sensitivity to NaCl or Sucrose in Female Rats

Maintenance diet alone did not affect the taste detectability of NaCl and sucrose in female rats as psychophysically assessed here. The measured thresholds for each group agree with those reported from other taste detection studies that used a similar testing paradigm [69,70] with intact male rats. However, one group reported a significant effect of HFD on taste thresholds for sucrose using conditioned taste aversion in a rat model of obesity [80]. In that study, male rats maintained on HFD had significantly lower sucrose thresholds than did chow-fed rats. In the evaluation of the disparity in outcomes, it is important to consider that there were important conceptual and methodological differences between the two studies. Conceptually speaking, the conditioned taste aversion procedure is designed to alter the hedonic evaluation of the conditioned stimulus. In contrast, the two-response operant taste detection task used here does not rely on the hedonic properties of the stimulus to drive responsiveness, rather the tastant serves as a cue to guide choice. In the former procedure, the sensory-discriminative and hedonic properties of the stimulus are conflated and difficult to disambiguate. Indeed, there is evidence to suggest that maintenance on a high-fat diet influences hedonic responsiveness to palatable stimuli. Some studies have found that rodents maintained on a high-fat diet display decreased motivated responsiveness to sugars relative to chow-fed animals [81,82,83]. Methodologically speaking, there were a number of issues that differed between the Sun et al. study [80] and ours. In the former, male rats were presented a series of daily 30-min two-bottle tests with descending sucrose (which served as the conditioned stimulus) concentrations and threshold was defined as the concentration at which the conditioned group did not statistically differ in sucrose preference from the unconditioned group. In our study, we tested female rats and took a more traditional psychophysical approach and determined thresholds for each animal individually by fitting curves to their concentration-performance functions and then derived EC_50_, which we operationally defined as threshold because it represents the inflection point and is optimally suited to discern lateral shifts in sensitivity along the concentration dimension. We then compared these EC_50_ values across groups. Moreover, in the two-response taste detection procedure, the tastant ingested on a given trial is limited to 10 licks, and the animal is forced to rapidly report the presence or absence of the stimulus, two methodological features that buttress the conclusion that behavior was under orosensory control.

### 4.2. RYGB Has Little Effect on Liminal Taste Sensitivity to NaCl or Sucrose

In humans, there are some reports of decreased detection or recognition thresholds to sucrose after RYGB [37,38], but such an outcome has not been universally observed [14] and in some cases, it appears that the change has more to do with weight loss than the surgery itself [36]. Here, notwithstanding a possible effect on NaCl sensitivity in the HFD condition, RYGB did not significantly influence the taste detection thresholds for NaCl or sucrose. These results suggest that reported changes in food selection and taste preference, at least in rodent models following RYGB, are not likely due to a surgery-induced influence on sensory-discriminative taste function, at least as measured by detection thresholds. The basis for the difference in outcomes between our study and those assessing patients after gastric bypass surgery might have several origins. First, there is always the possibility that this represents a fundamental species difference. Second, it is possible that the exact nature of the psychophysical procedure used to assess thresholds across studies may have an impact on whether surgery-induced changes in perithreshold taste sensitivity are observed. Third, the exact timing of the threshold determination postsurgery may be critical. In our study, we tested rats when they were no longer in their weight loss phase, whereas, in humans, all the studies were conducted on patients within 6 months after surgery when body weight is still changing. These are not mutually exclusive possibilities.

### 4.3. Postprandial Circulating Levels of Leptin Were Not Related to Sucrose (or NaCl) Taste Detection Thresholds

After RYGB, the rats in our study displayed the expected decrease in body weight and fat mass, and the predicted accompanying changes in circulating postprandial plasma levels of GLP-1 (increased) and leptin (decreased). Leptin is thought to selectively decrease behavioral and neural responsiveness to sweeteners and the leptin receptor (Ob-R) is expressed in taste buds [84]. A mutant strain of obese mice (db/db) lacking a normal functioning leptin receptor (Ob-R) displays enhanced preference for, and chorda tympani nerve (innervating taste buds on the anterior tongue) responses to, sweeteners including sucrose [84,85]. The administration of leptin decreases such responses in wildtype mice but not in db/db mice. Indeed, it has been shown that, in humans without obesity, sucrose recognition thresholds covaried with diurnal changes in leptin levels such that sensitivity was higher in the morning when leptin levels were lower, and they were lower in the evening when leptin levels were higher; recognition thresholds for other nonsweet tastants did not vary in this manner [86]. Interestingly, in humans with obesity, the positive relationship between plasma leptin and sucrose recognition thresholds disappears and is replaced by a negative one such that higher leptin levels are associated with increased sucrose sensitivity [86]; this outcome was attributed to leptin resistance. It appears that the effect of leptin on peripheral taste processes saturates at plasma levels ~15–20 ng/mL [84,86]. However, for those rats from which we were able to sample blood at the end of the experiment, all but two (which were in the SHAM HFD group) had postprandial plasma leptin levels that were below ~15 ng/mL, and there was no significant relationship between leptin levels and sucrose (or NaCl) EC_50_ for either RYGB or SHAM rats, nor for the two types of groups combined. Moreover, while RYGB significantly decreased plasma leptin levels in the DC and Chow groups relative to their SHAM counterparts, these groups did not differ in their sucrose (or NaCl) sensitivity. One important consideration is that we only assessed plasma leptin levels at the end of the experiment and not during the sensory test sessions. While this limitation may have influenced the variance of leptin levels within groups affecting correlations with behavior, it is unlikely to have affected the differences between groups. It should be noted that a leptin-related decrease in behavioral or taste nerve responsiveness to sweeteners has not always been observed in mice, even when leptin levels are below 10 ng/mL [87,88]. Thus, whether circulating leptin affects taste sensitivity still remains an open question; we did not see evidence for it as assessed here.

### 4.4. Caveats Regarding Lack of Effects of Diet and RYGB on Taste Sensitivity

Although we found no effect of diet or RYGB on sucrose detection thresholds and an equivocal effect at most on NaCl detectability in our rat model, this does not mean these manipulations cannot or do not have an impact on taste processing. It is important to stress that we only measured detection thresholds here. It is possible that other measures of taste sensitivity including the recognition threshold, which is the lowest concentration at which the quality of the stimulus can be identified, or suprathreshold intensity measurements, might be influenced by RYGB or diet. It is, therefore, plausible to consider that, while diet and RYGB did not influence detection threshold, these manipulations might exert an influence on recognition threshold and/or perceived suprathreshold intensity. The latter measure, which involves psychophysical scaling procedures such as magnitude estimation, can only be applied with humans. That said, the use of the two-response taste detection procedure to assess detection thresholds has proven to be exceptionally sensitive to genetic, pharmacological, and anatomical manipulations of the peripheral and central gustatory system in rodent models [69,70,89,90,91,92,93,94,95].

It is important to stress that the failure of the maintenance diet alone to exert a significant effect on threshold sensitivity to NaCl and sucrose in female rats is a conclusion specific to the conditions tested here. The HFD that we used was purchased from Research Diets (D12451), and it was compared to common laboratory chow (Purina 5001). These diets differed on many different characteristics, and it is possible that changes in the formulations used to compose these foods could result in different outcomes. Indeed, there is growing evidence that the presence and concentration of specific components in diets can impact neural and behavioral responsiveness to taste, including effects on taste detection thresholds assessed with a task similar to the one used here, through their action on salivary protein expression [94,96].

### 4.5. The Numbers of Fungiform Papillae and Fungiform and Circumvallate Taste Buds Were Unaffected by HFD Maintenance and RYGB

There is growing evidence suggesting that, at least under certain conditions, exposure to an HFD can decrease taste bud number in the CV of some strains of mice [16] and rats [18] and can also decrease the density of fungiform papillae, which house taste buds, in the anterior tongue of rodents [18,79]. Likewise, fungiform papillae density was found to be negatively correlated with an anthropometric marker of obesity in humans [79]. Expression of inflammatory and taste-signaling markers in taste buds has also been reported to be altered by HFD exposure in mice [16,79] and related to obesity in humans [97]. Here, there was no evidence that chronic exposure to HFD (nor RYGB) influences the quantity of taste buds in female rats. Of course, we cannot dismiss the possibility that different maintenance diets or diet exposure times may have revealed effects. It is also possible that either maintenance diet or RYGB could have influences on the relative numbers of different taste bud cell types or taste receptor cell function. There is evidence that HFD exposure also alters some signaling properties in taste receptor cells [17]. However, if such events were occurring in our rats, they did not influence psychophysically assessed taste detection thresholds for sucrose and NaCl.

## 5. Concluding Remarks

Overall, despite the substantial effects that HFD maintenance and RYGB have on body mass and composition, circulating levels of key feeding-related hormones, and energy intake, it has little, if any, effect on taste sensitivity to the essential electrolyte sodium and the cardinal sugar sucrose, at least as assessed at the limens of detectability. These findings suggest that reported changes in food choice and preference associated with RYGB, at least in rats, are not likely driven by changes in very basic sensory functions of the gustatory system. The fact that liminal sensitivity to taste compounds tested here was refractory to our dietary and bariatric manipulations, both of which have salient physiological consequences, suggests that basic sensory-discriminative taste function is relatively immune to such challenges, perhaps to ensure that foods and fluids in the environment can be reliably detected and identified regardless of external or internal conditions. Such basic sensory information can then be acted on by other neural circuits to appropriately modify an animal’s behavior in an adaptive manner.

## Figures and Tables

**Table 1:** Timeline overview.

Phase	Duration
Acclimation ^1^	10 weeks
Surgery and Recovery	4–6 weeks
Training ^2^	10 weeks
Spout	6–15 sessions
Side	6–10 sessions
Alternation	4 sessions
Random I	7–14 sessions
Random II	2–15 sessions
Test NaCl	6 weeks
Train Sucrose	1 week
Test Sucrose ^3^	7 weeks
Stimulus Control Test	1 session/animal
EchoMRI ^4^	1 day
Tissue Collection ^5^	2 weeks

^1^ Acclimation to diet; one night of acclimation to the post-surgical housing and diet conditions. ^2^ Phase 2 had 5 weeks of an additional “holding period” during training due to COVID-19. ^3^ An unsuccessful effort to train rats to reliably detect linoleic acid was attempted in Phase 1 after sucrose testing (see *Stimuli* for more details). ^4^ Only rats from Phase 2. ^5^ “Tissue Collection”: Tongue tissue, fat pads, EchoMRI, and blood plasma where applicable (see text).

**Table 2:** Group sizes for statistical analysis.

	HFD	DC	Chow
NaCl Testing	R = 5; S = 7	R = 9; S = 6	R = 8; S = 7
Sucrose Testing	R = 5; S = 7	R = 9; S = 6	R = 8; S = 7
EchoMRI	R = 1; S = 4	R = 4; S = 4	R = 6; S = 4
Fat Pads	R = 3; S = 5	R = 4; S = 4	R = 6; S = 4
Anterior Taste Pores	R = 5; S = 7	R = 8; S = 6	R = 8; S = 7
CV Taste Pores	R = 4; S = 7	R = 6; S = 5	R = 7; S = 7
GLP-1 ELISA *	R = 2; S = 6	R = 6; S = 6	R = 6; S = 7
Leptin ELISA	R = 2; S = 6	R = 3; S = 6	R = 6; S = 7

* Enzyme-linked Immunosorbent Assay.

**Table 3:** Statistical analysis from 3-way, repeated measures ANOVA comparing body weight during critical testing periods (Figure 3 top). Significant values are noted by **bold** text.

	Acclimation	Recovery	Detection Training	NaCl Testing	Sucrose Training	Sucrose Testing
Surg	F (1,36) = 2.97, *p* = 0.094	**F (1,36) =** **21.35,** ***p* < 0.001**	**F (1,36) =** **24.50,** ***p* < 0.001**	**F (1,36) =** **25.90,** ***p* < 0.001**	**F (1,36) =** **30.76,** ***p* < 0.001**	**F (1,36) =** **30.76,** ***p* < 0.001**
Diet	**F (2,36) =** **21.58,** ***p* < 0.001**	**F (2,36) =** **21.95,** ***p* < 0.001**	**F (2,36) =** **9.59,** ***p* < 0.001**	**F (2,36) =** **13.23,** ***p* < 0.001**	**F (2,36) =** **12.82,** ***p* < 0.001**	**F (2,36) =** **12.82,** ***p* < 0.001**
Time	**F (9,324) =** **234.55,** ***p* < 0.001**	**F (25,900) = 6.83, *p* < 0.001**	**F (8,288) =** **7.62,** ***p* < 0.001**	**F (5,180) =** **18.24,** ***p* < 0.001**		F (6,216) = 1.49, *p* = 0.18
Surg × Diet	F (2,36) = 2.74, *p* = 0.078	F (2,36) = 0.71, *p* = 0.50	F (2,36) = 2.33, *p* = 0.11	F (2,36) = 2.30, *p* = 0.12	F (2,36) = 1.86, *p* = 0.17	F (2,36) = 1.86, *p* = 0.17
Surg × Time	F (9,324) = 0.54, *p* = 0.83	**F (25,900) =** **15.94,** ***p* < 0.001**	F (8, 288) = 1.45, *p* = 0.18	**F (5,180) =** **8.19,** ***p* < 0.001**		F (6,216) = 1.20, *p* = 0.31
Diet × Time	**F (18,324)** **5.91,** ***p* < 0.001**	**F (50,900) =** **14.65,** ***p* < 0.001**	**F (16,288) =** **2.49,** ***p* < 0.001**	F (10,180) = 0.72, *p* = 0.71		F (12,216) = 0.77, *p* = 0.68
Surg × Diet × Time	F (18,324) = 1.04, *p* = 0.41	**F (50,900) =** **6.84,** ***p* < 0.001**	F (16,288) = 0.71, *p* = 0.79	F (10,180) = 0.55, *p* = 0.89		**F (12,216) =** **2.03,** ***p* = 0.023**

**Table 4:** Statistical analysis from 3-way, repeated measures ANOVA comparing percentage change body weight during the critical testing periods (Figure 3, bottom). Significant values are noted by **bold** text.

	Acclimation	Recovery	Detection Training	NaCl Testing	Sucrose Training	Sucrose Testing
Surg	F (1,36) = 0.22, *p* = 0.65	**F (1,36) =** **74.50,** ***p* < 0.001**	**F (1,36) =** **36.14,** ***p* < 0.001**	**F (1,36) =** **39.42,** ***p* < 0.001**	**F (1,36) =** **33.99,** ***p* < 0.001**	**F (1,36) =** **43.22,** ***p* < 0.001**
Diet	**F (2,36) =** **13.29,** ***p* < 0.001**	**F (2,36) =** **7.60,** ***p* = 0.002**	**F (2,36) =** **19.81,** ***p* < 0.001**	**F (2,36) =** **15.77,** ***p* < 0.001**	**F (2,36) =** **9.54,** ***p* < 0.001**	**F (2,36) =** **12.27,** ***p* < 0.001**
Time	**F (9,324) =** **224.98,** ***p* < 0.001**	**F (25,900) =** **6.64,** ***p* < 0.001**	**F (8,288) =** **7.75,** ***p* < 0.001**	**F (5,180) =** **8.95,** ***p* < 0.001**		F (6,216) = 1.65, *p* = 0.13
Surg × Diet	F (2,36) = 2.34, *p* = 0.11	**F (2,36) =** **3.60,** ***p* = 0.037**	**F (2,36) =** **13.87,** ***p* < 0.001**	**F (2,36) =** **10.69,** ***p* < 0.001**	**F (2,36) =** **5.74,** ***p* = 0.007**	**F (2,36) =** **8.36,** ***p* = 0.001**
Surg × Time	F (9,324) = 0.43, *p* = 0.92	**F (25, 900) =** **16.30,** ***p* < 0.001**	F (8, 288) = 1.58, *p* = 0.13	**F (5,180) =** **4.62,** ***p* = 0.001**		F (6,216) = 1.23, *p* = 0.29
Diet × Time	**F (18, 324)** **= 5.46,** ***p* < 0.001**	**F (50, 900) =** **15.93,** ***p* < 0.001**	**F (16, 288) =** **2.24,** ***p* = 0.004**	F (10,180) = 0.72, *p* = 0.71		F (12,216) = 0.90, *p* = 0.55
Surg × Diet × Time	F (18, 324) = 1.06, *p* = 0.39	**F (50, 900) =** **6.03,** ***p* < 0.001**	F (16, 288) = 0.72, *p* = 0.79	F (10,180) = 0.94, *p* = 0.50		**F (12,216) =** **1.87,** ***p* = 0.039**

**Table 5:** Statistical analysis comparing body composition measures. For paired comparisons, uncorrected *p*-values are depicted; those that survived Bonferroni corrections are **bolded**. ANOVA analyses compared the overall effect of diet on the body composition measures between SHAM groups. Significant ANOVAs are also **bolded**.

	Between Chow	Between Diet Change	Between RYGB	Between SHAM			
	RYGB vs. SHAM	RYGB vs. SHAM	Chow vs. Diet Change	ANOVA	Chow vs. Diet Change	Chow vs. High-Fat Diet	Diet Change vs. High-Fat Diet
Total Fat Pad Weight	**t(8) = 7.16,** ***p* < 0.001**	**t(6) = 6.89,** ***p* < 0.001**	t(8) = 1.89, *p* = 0.096	**F (2,10) = 5.01,** ***p*= 0.031**	t(6) = 0.53, *p* = 0.62	t(7) = 2.21, *p* = 0.063	t(7) = 2.33, *p* = 0.053
Retro Peritoneal Fat Pad	**t(8) = 6.99,** ***p* < 0.001**	**t(6) = 8.48,** ***p* < 0.001**	t(8) = 1.76, *p* = 0.12	**F (2,10) = 4.76,** ***p* = 0.035**	t(6) = 0.41, *p* = 0.69	t(7) = 2.23, *p* = 0.061	t(7) = 2.17, *p* = 0.066
Gonadal Fat Pad	**t(8) = 6.10,** ***p* < 0.001**	**t(6) = 3.93,** ***p* = 0.008**	t(8) = 1.69, *p* = 0.13	F (2,10) = 2.87, *p* = 0.10	t(6) = 1.0, *p* = 0.35	t(7) = 1.52, *p* = 0.17	t(7) = 1.95, *p* = 0.093
Perirenal Fat Pad	**t(8) = 6.30,** ***p* < 0.001**	**t(6) = 5.43,** ***p* < 0.001**	t(8) = 1.70, *p* = 0.13	**F (2,10) = 5.53,** ***p* = 0.024**	t(6) = 1.80, *p* = 0.12	t(7) = 2.07, *p* = 0.077	t(7) = 2.67, *p* = 0.032
Body Weight on MRI Scan	**t(8) = 4.83,** ***p* = 0.001**	**t(6) = 3.63,** ***p* = 0.011**	t(8) = 0.23, *p* = 0.82	F (2,9) = 2.67, *p* = 0.12	t(6) = 0.09, *p* = 0.93	t(6) = 1.67, *p* = 0.15	t(6) = 1.69, *p* = 0.14
%Fat Mass	**t(8) = 3.23,** ***p* = 0.012**	**t(6) = 5.41,** ***p* = 0.002**	t(8) = 1.91, *p* = 0.093	**F (2,9) = 4.98,** ***p* = 0.035**	t(6) = 0.11, *p* = 0.91	t(6) = 2.22, *p* = 0.068	t(6) = 2.34, *p* = 0.058
%Lean Mass	**t(8) = 3.03,** ***p* = 0.016**	**t(6) = 4.40,** ***p* = 0.005**	t(8) = 1.59, *p* = 0.15	**F (2,9) = 4.71,** ***p* = 0.040**	t(6) = 0.24, *p* = 0.82	t(6) = 2.22, *p* = 0.068	t(6) = 2.22, *p* = 0.068
%Bone Mass	t(8) = 0.76, *p* = 0.47	t(6) = 0.37, *p* = 0.72	t(8) = 0.23, *p* = 0.83	F (2,9) = 2.25, *p* = 0.16	t(6) = 1.05, *p* = 0.34	t(6) = 1.17, *p* = 0.29	t(6) = 1.98, *p* = 0.096

**Table 6:** Group means (and standard errors) for each of the parameters included in the logistic function that was used to generate individual curve fits for NaCl and sucrose testing.

		Chow		Diet Change		High-Fat Diet	
		RYGB	SHAM	RYGB	SHAM	RYGB	SHAM
NaCl Curve Parameters	Asymptote (A)	0.95 (0.009)	0.97 (0.007)	0.97 (0.008)	0.94 (0.008)	0.97 (0.007)	0.97 (0.010)
	Threshold (EC_50_)	−2.39 (0.16)	−2.72 (0.11)	−2.61 (0.089)	−2.58 (0.18)	−2.86 (0.098)	−2.41 (0.14)
	Slope (B)	−1.11 (0.12)	−1.12 (0.09)	−1.50 (0.53)	−2.14 (1.21)	−1.04 (0.14)	−0.83 (0.089)
	R-squared	0.93 (0.022)	0.93 (0.013)	0.91 (0.019)	0.93 (0.016)	0.95 (0.008)	0.87 (0.015)
Sucrose Curve Parameters	Asymptote (A)	0.95 (0.003)	0.98 (0.009)	0.96 (0.009)	0.96 (0.009)	0.96 (0.01)	0.95 (0.013)
	Threshold (EC_50_)	−1.93 (0.044)	−1.89 (0.068)	−1.89 (0.045)	−2.04 (0.11)	−1.89 (0.069)	−1.83 (0.053)
	Slope (B)	−2.47 (0.53)	−2.23 (0.55)	−1.81 (0.13)	−2.09 (0.64)	−1.55 (0.17)	−1.60 (0.27)
	R-squared	0.93 (0.019)	0.92 (0.031)	0.94 (0.014)	0.88 (0.038)	0.95 (0.018)	0.92 (0.026)

**Table 7:** Statistical analysis comparing each of the three parameters included in the logistics function that was used to generate individual curve fits for NaCl and sucrose testing. For paired comparisons, uncorrected *p*-values are depicted; those that survived Bonferroni corrections are **bolded**. Significant ANOVAs are also **bolded**.

		Surgery	Diet	Interaction
NaCl Curve Parameters	Asymptote (A)	F (1,36) = 0.61, *p* = 0.44	F (2,36) = 1.26, *p* = 0.30	F (2,36) = 3.11, *p* = 0.057
	Threshold (EC_50_)	F (1,36) = 0.19, *p* = 0.67	F (2,36) = 0.17, *p* = 0.85	**F (2,36) =** **4.06,** ***p* = 0.026**
	Between RYGB		F (2,19) = 3.04, *p* = 0.072	
	Between SHAM		F (2,17) = 1.27, *p* = 0.31	
	Between Chow	t (13) = 1.68, *p* = 0.12		
	Between DC	t (13) = 0.16, *p* = 0.88		
	Between HFD	t (10) = 2.46, *p* = 0.034		
	Slope (B)	F (1,36) = 0.12, *p* = 0.73	F (2,36) = 1.61, *p* = 0.21	F (2,36) = 0.36, *p* = 0.70
Sucrose Curve Parameters	Asymptote (A)	F (1,36) = 0.47, *p* = 0.50	F (2,36) = 0.52, *p* = 0.60	F (2,36) = 1.56, *p* = 0.22
	Threshold (EC_50_)	F (1,36) = 0.10, *p* = 0.75	F (2,36) = 1.27, *p* = 0.29	F (2,36) = 1.50, *p* = 0.24
	Slope (B)	F (1,36) = 0.006, *p* = 0.94	F (2,36) = 1.56, *p* = 0.22	F (2,36) = 0.19, *p* = 0.83

**Table 8:** Statistical analysis comparing outcomes of the hormone response tests. For paired comparisons, uncorrected *p*-values are depicted; those that survived Bonferroni corrections are **bolded**. Significant ANOVAs are also **bolded**.

	Chow	Diet Change	Between RYGB	Between SHAM			
	RYGB vs. SHAM	RYGB vs. SHAM	Chow vs. Diet Change	ANOVA	Chow vs. Diet Change	Chow vs. High-Fat Diet	Diet Change vs. High-Fat Diet
Body Weight	**t (11) = 4.21,** ***p* = 0.001**	**t (10) = 4.58,** ***p* = 0.001**	t (10) = 0.53, *p* = 0.61	**F (2,16) = 11.61,** ***p* = 0.001**	t (11) = 0.31, *p* = 0.76	**t (11) = 3.92,** ***p* = 0.002**	**t (10) = 3.52,** ***p* = 0.006**
Caloric Intake	**t (11) = 9.089,** ***p* < 0.001**	**t (10) = 3.52,** ***p* = 0.006**	t (10) = 0.40, *p* = 0.70	**F (2,16) = 9.53,** ***p* = 0.002**	t (11) = 2.79, *p* = 0.018	**t (11) = 5.79,** ***p* < 0.001**	t (10) = 0.90, *p* = 0.39
GLP-1	**t (11) = 3.46,** ***p* = 0.005**	t (10) = 2.68, *p* = 0.023	t (10) = 0.89, *p* = 0.40	F (2,16) = 1.63, *p* = 0.23	t (11) = 1.29, *p* = 0.22	t (11) = 1.61, *p* = 0.14	t (10) = 0.22, *p* = 0.83
Leptin	**t (11) = 3.68,** ***p* = 0.004**	t (7) = 3.036, *p* = 0.019	t (7) = 1.82, *p* = 0.11	**F (2,16) = 6.81,** ***p* = 0.007**	t (11) = 0.40, *p* = 0.70	**t (11) = 2.84,** ***p* = 0.016**	t (10) = 2.46, *p* = 0.033

## Data Availability

The data that support the findings of this study are available from the corresponding author upon reasonable request.

## Supplementary Materials

The following supporting information can be downloaded at: https://www.mdpi.com/article/10.3390/nu14040877/s1, Figure S1: Scatter plots for significantly correlated measures. Overall Pearson’s R and significant *p*-values are noted within the figure panels (a–d; top row) and significant correlations within the SHAM group (e–f; bottom row) or RYGB group (g; bottom row); corresponding values from within surgery comparisons are in Tables S1 and S2. RYGB animals are represented by open circles and SHAMs by filled circles. Brown symbols represent the Chow group; blue, the diet change; pink, the high-fat diet. Table S1: Overall and within-surgery correlations between taste thresholds and fat mass, hormones, and taste pores. Significant *p*-values are **bold**. Table S2: Overall and within-surgery correlations conducted for key measures during hormone and body composition tests. Significant *p*-values are **bold**.
