# Supplementary material for: The Influence of Roux-en-Y Gastric Bypass and Diet on NaCl and Sucrose Taste Detection Thresholds and Number of Circumvallate and Fungiform Taste Buds in Female Rats
Source: Nutrients. 2022 Feb 19;14(4):877. doi: 10.3390/nu14040877 (PMC8880222; doi:10.3390/nu14040877)
Supplement: Supplementary file 1 [file nutrients-14-00877-s001.zip › nutrients-1579075-supplementary.pdf]

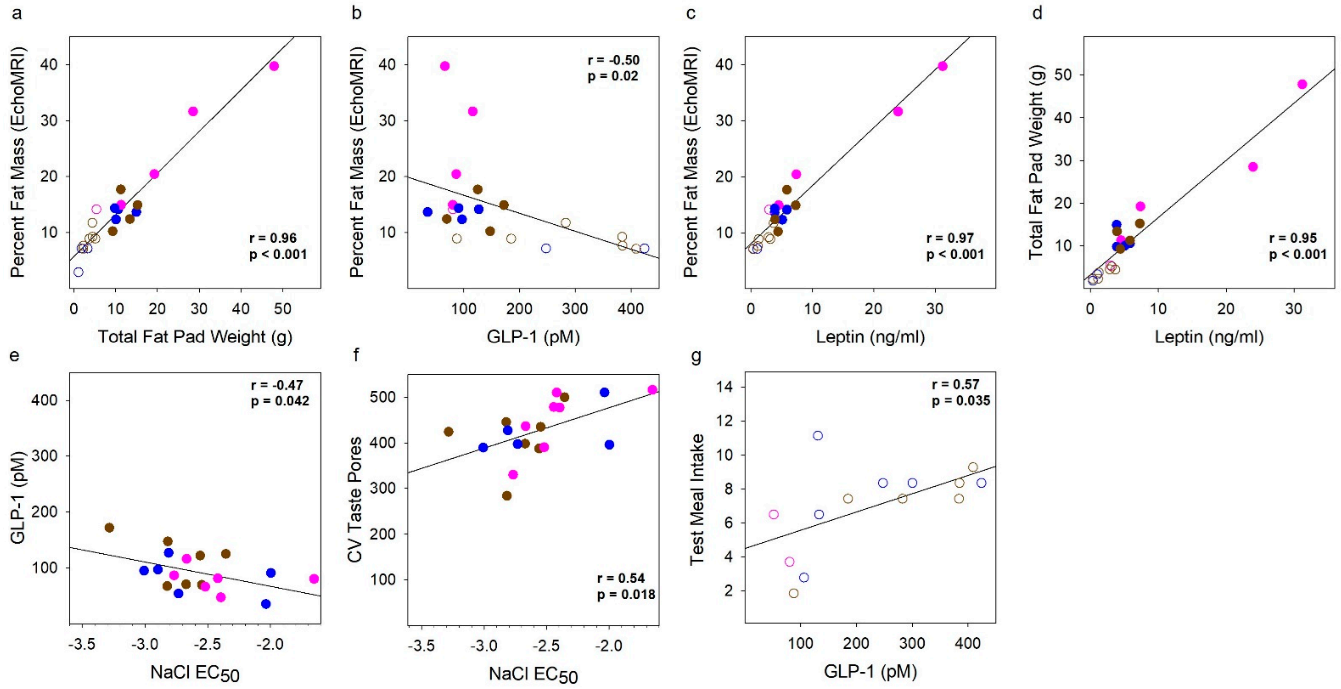

Figure S1. Scatter plots for significantly correlated measures. Overall Pearson's R and significant p-values are noted within the figure panels (a-d; top row) and significant correlations within the SHAM group (e-f; bottom row) or RYGB group (g; bottom row); corresponding values from within surgery comparisons are in Tables S1 and S2. RYGB animals are represented by open circles and SHAMs by filled circles. Brown symbols represent the chow group; blue, the diet change; pink, the high-fat diet.

Table S1. Overall and within-surgery correlations between taste thresholds and fat mass, hormones, and taste pores. Significant p-values are **bold.**

Correlation	r	*p*
NaCl EC_{50} v Fat Pad	-0.114	0.58
RYGB	-0.33	0.26
SHAM	-0.033	0.92
NaCl EC_{50} v Fat Mass(MRI)	-0.07	0.76
RYGB	-0.039	0.91
SHAM	-0.028	0.93
NaCl EC_{50} v GLP-1	0.080	0.66
RYGB	0.23	0.44
**SHAM**	**-0.471**	**0.042**
NaCl EC_{50} v Leptin	-0.037	0.84
RYGB	0.34	0.31
SHAM	0.006	0.98
NaCl EC_{50} v Fungiform Pores	0.21	0.20
RYGB	0.051	0.83
SHAM	0.34	0.14
NaCl EC_{50} v CV Pores	0.32	0.056
RYGB	0.11	0.67
**SHAM**	**0.54**	**0.018**
Sucrose EC_{50} v Fat Pad	-0.054	0.79
RYGB	-0.028	0.93
SHAM	-0.21	0.49
Sucrose EC_{50} v Fat Mass(MRI)	0.002	0.99
RYGB	0.094	0.78
SHAM	-0.12	0.72
Sucrose EC_{50} v GLP-1	0.11	0.53
RYGB	0.033	0.91
SHAM	0.43	0.065
Sucrose EC_{50} v Leptin	0.22	0.23
RYGB	0.22	0.51
SHAM	0.29	0.23
Sucrose EC_{50} v FungiformPores	-0.015	0.93
RYGB	0.20	0.39
SHAM	-0.13	0.60
Sucrose EC_{50} v CV Pores	-0.097	0.57
RYGB	-0.38	0.13
SHAM	-0.085	0.73

Table S2. Overall and within-surgery correlations conducted for key measures during hormone and body composition tests. Significant p-values are **bold.**

Correlation	r	*p*
GLP-1 v Fat Pad	**-0.52**	**0.016**
RYGB	-0.61	0.081
SHAM	-0.27	0.40
**GLP-1 v Percent Fat Mass** (MRI)	**-0.50**	**0.02**
RYGB	-0.60	0.089
SHAM	-0.19	0.56
GLP-1 v Test Meal Intake	-0.31	0.077
**RYGB**	**0.57**	**0.035**
SHAM	0.31	0.20
GLP-1 v Leptin	-0.33	0.073
RYGB	-0.13	0.70
SHAM	-0.086	0.73
GLP-1 v Fungiform Pores	0.038	0.83
RYGB	0.29	0.32
SHAM	-0.32	0.18
GLP-1 v CV Pores	-0.084	0.67
RYGB	0.096	0.75
SHAM	-0.34	0.17
**Leptin v Fat Pad**	**0.95**	**< 0.001**
**RYGB**	**0.89**	**0.001**
**SHAM**	**0.96**	**< 0.001**
**Leptin v Percent Fat Mass (MRI)**	**0.97**	**< 0.001**
**RYGB**	**0.77**	**0.016**
**SHAM**	**0.97**	**< 0.001**
Leptin v Fungiform Pores	-0.073	0.70
RYGB	0.48	0.14
SHAM	-0.19	0.43
Leptin v CV Pores	0.13	0.51
RYGB	-0.16	0.67
SHAM	0.089	0.73
**Fat Pad v Percent Fat (MRI)**	**0.96**	**< 0.001**
**RYGB**	**0.85**	**0.002**
**SHAM**	**0.96**	**< 0.001**
Percent Fat Mass v Fungiform Pores	0.12	0.58
RYGB	0.31	0.39
SHAM	-0.009	0.98
Percent Fat Mass v CV Pores	0.073	0.77
RYGB	0.12	0.77
SHAM	-0.053	0.88
